# Southeast Texas Medical Association

**Published:** 1897-05

**Authors:** 


### The Southeast Texas Medical Association.

Met in regular session at the City Hall at Beaumont, on the 6th of April ult., with the largest attendance known in the history of the organization.

In the absence of the president and vice-president Dr. J. S. Price was elected to preside over the meeting.

After the reading of the minutes by Secretary Calhoun, an interesting case of several years' standing, luxation of femur with formation of false joint was examined and discussed by all present.

Dr. B. F. Calhoun related a case of angioneurotic œdema occurring in an elderly lady at Sabine Pass, when upon exposure to cold, the ears and hands became extremely œdematous.

The doctor called attention to the differential diagnosis between this affection and urticaria, and also mentioned the scarcity of literature on the subject.

Dr. Sholars, of Orange, reported a case of contraction of conjugate diameter of the brim of pelvis, which demonstrated the risk some women are willing to undergo in order that they may clasp to their bosom a living child. The patient in question had lost by craniatomy five children in consequence of her extremely deformed pelvis.

Dr. Sanders, of Beaumont, called attention to the difficulties of managing obstetrical cases among certain classes of Mexicans.

Dr. Price gave his experience in the treatment of a case of mucous collitis in which the bowel continued to shed its lining for three months, at times casts of the entire bowel would pass. Reference was made to the relation of the nervous system as the main etiological factor in this class of patients.

Those present were as follows: Doctors Buttler, Seastrunk, Sr., Seastrunk, Jr., Sholars, Stone, Yates, Bean, Stephens,

Masterson, Calhoun, Cunningham, Sanders, Nash, Stovall, Haines and Price, A. N. Perkins, Sabine Pass.

Beaumont was selected as the next place of meeting on the second Tuesday in July.

